# Impact of Calcium Signaling during Infection of *Neisseria meningitidis* to Human Brain Microvascular Endothelial Cells

**DOI:** 10.1371/journal.pone.0114474

**Published:** 2014-12-02

**Authors:** Tauseef M. Asmat, Tobias Tenenbaum, Ann-Beth Jonsson, Christian Schwerk, Horst Schroten

**Affiliations:** 1 Department of Pediatrics, Pediatric Infectious Diseases, Medical Faculty Mannheim, Heidelberg University, Mannheim, Germany; 2 Department of Molecular Biosciences, The Wenner-Gren Institute, Stockholm University, Stockholm, Sweden; Cinvestav-IPN, Mexico

## Abstract

The pili and outer membrane proteins of *Neisseria meningitidis* (meningococci) facilitate bacterial adhesion and invasion into host cells. In this context expression of meningococcal PilC1 protein has been reported to play a crucial role. Intracellular calcium mobilization has been implicated as an important signaling event during internalization of several bacterial pathogens. Here we employed time lapse calcium-imaging and demonstrated that PilC1 of meningococci triggered a significant increase in cytoplasmic calcium in human brain microvascular endothelial cells, whereas PilC1-deficient meningococci could not initiate this signaling process. The increase in cytosolic calcium in response to PilC1-expressing meningococci was due to efflux of calcium from host intracellular stores as demonstrated by using 2-APB, which inhibits the release of calcium from the endoplasmic reticulum. Moreover, pre-treatment of host cells with U73122 (phospholipase C inhibitor) abolished the cytosolic calcium increase caused by PilC1-expressing meningococci demonstrating that active phospholipase C (PLC) is required to induce calcium transients in host cells. Furthermore, the role of cytosolic calcium on meningococcal adherence and internalization was documented by gentamicin protection assay and double immunofluorescence (DIF) staining. Results indicated that chelation of intracellular calcium by using BAPTA-AM significantly impaired PilC1-mediated meningococcal adherence to and invasion into host endothelial cells. However, buffering of extracellular calcium by BAPTA or EGTA demonstrated no significant effect on meningococcal adherence to and invasion into host cells. Taken together, these results indicate that meningococci induce calcium release from intracellular stores of host endothelial cells via PilC1 and cytoplasmic calcium concentrations play a critical role during PilC1 mediated meningococcal adherence to and subsequent invasion into host endothelial cells.

## Introduction

*Neisseria meningitidis* (*N. meningitidis*) are Gram-negative human-specific pathogens and the leading cause of meningitis and sepsis worldwide [1], [2]. Since meningococci can be transmitted by direct contact from person to person the carriage rate in healthy individuals is very high (10 to 40%), and the human upper respiratory tract serves as a reservoir for meningococci [2]–[4]. Despite high carriage rates, only few bacteria can invade the blood stream to cause septicemia or disrupt the blood-brain barrier (BBB) to cause meningitis [2]. Lack of appropriate *in vivo* and *in vitro* models has contributed to the incomplete understanding of meningococcal pathogenesis, which in turn leads to high morbidity and mortality (10 to 50%) in people suffering from meningococcal septicemia [5]. In order to cause disease, the pathogens have to survive in hostile environment, have to overcome the host cell defense and have to breach different barriers like the BBB [6]. Bacterial pathogens like meningococci, *Escherichia coli* K1 (*E. coli*), *Streptococcus agalactiae (S. agalactia), S. pneumoniae* and *Haemophilus influenza type b* (*H. influenza*) are equipped with different virulence factors and have also evolved different strategies to cross the BBB to cause meningitis in people of different age groups [7], [8]. Among meningococcal virulence factors, pili serve as primary adhesins and thus initiate the infectious process of meningococci by providing initial attachment and promoting adhesion to host cells [9], [10].

Meningococcal pili belong to type IV class of pili and these pili play a critical role during adherence and subsequent invasion of several bacterial pathogens to host cells [11]. Pilin (PilE) and PilC are two major components of neisserial pili. Type IV pili primarily consist of monomeric PilE protein and are extended from bacterial surface as a filamentous structure [12]. Besides adhesion, cytotoxicity, twitching motility, DNA transformation and auto-aggregation are some peculiar attributes of meningococcal pili [13], [14]. PilC acts as a tip-associated bacterial adhesion [15]. Most pathogenic *N. meningitidis* and *Neisseria gonorrhoeae* (*N. gonorrhoeae*) express two PilC alleles, *pilC1* and *pilC2*, and these two variants are independently expressed from separate loci and regulated distinctly [16]–[19].

Type IV pili-mediated meningococcal interaction with host cells leads to cytoskeleton rearrangements and modulates the host cell signaling cascades [20]. During this interaction, eukaryotic membrane-associated proteins such as Intercellular Adhesion Molecule 1(ICAM-1), Epidermal growth factor receptor (EGFR), CD44 and CD46 get accumulated and lead to actin polymerization [21]–[24]. CD46 is a complement regulator, expressed by almost all human cell types except erythrocytes and it has been reported to be involved during interaction of pathogenic *Neisseria* with human host cells [25], [26]. During infection of *N. gonorrhoeae* CD46 gets phosphorylated [27], [28] to induce cell signaling and transient release of calcium (Ca^2+^) from intracellular stores in human cervical carcinoma epithelial cells (ME180) [29]. Calcium signaling plays a key role in cell proliferation, gene expression, vascular contraction, and enzyme secretion [30]. Moreover, virulence factors of several bacterial pathogens like *Salmonella typhimurium* (*S. typhimurium*) [31], [32], *Listeria monocytogenes* (*L. monocytogenes*) [33], [34], *Campylobacter jejuni* (*C. jejuni*) [35], *Pseudomonas aeruginosa (P.aeruginosa)* [36] *Kingella kingae* (*K. kingae*) [37] and *S. pneumoniae* [38], [39] have been reported to induce calcium influx in host cells, and such modulation of calcium signaling facilitates the bacterial adherence to and subsequent internalization into their respective host cells. Bacterial toxins, like hemolysin A from *Staphylococcus aureus* (*S. aureus*), have been shown to induce calcium transients in host epithelial cells [40]. However, the role of calcium mobilization during meningococcal interaction with human endothelial cells and its subsequent effect on meningococcal adherence to host endothelial cells has not been addressed yet.

In this study, we analyzed the role of calcium signaling during meningococcal interaction with Human Brain Microvascular Endothelial Cells (HBMEC) in detail. During the last two decades, HBMEC have emerged as a prime model to study the impact of meningitis causing pathogens on the human blood-brain barrier [41]–[47]. Our study demonstrated that meningococci induce calcium efflux from intracellular stores of host endothelial cells by PilC1 and this release of calcium was dependent on phospholipase C. Furthermore, calcium release from intracellular stores was critical for efficient adherence and subsequent invasion of meningococci into HBMEC.

## Materials and Methods

### Bacterial strains and culture conditions

The *N. meningitidis* serogroup C strains FAM20 (WT), FAM20.1 (Δ*pilC1*) and FAM20.2 (Δ*pilC2*) were grown overnight at 37°C and 5% CO_2_ on chocolate agar plates supplemented with 1% Polyvitox (Oxoid, Wesel, Germany). FAM20 and its isogenic mutants of *pilC1* (FAM20.1) and *pilC2* (FAM20.2) have previously been described [48]. *Lactococcus lactis (L. lactis)* MG1363 were cultured statically at 30°C in M17 broth (Oxoid) supplemented with 0.5 % glucose (GM17) to mid-exponential phase or grown on GM17 agar plates (Oxoid).

### Chemicals and reagents

1,2-bis-(o-Aminophenoxy)-ethane-N,N,N’,N’-tetraacetic acid, tetraacetoxymethyl ester (BAPTA-AM) was purchased from Calbiochem (Darmstadt, Germany). Trypsin (without EDTA) was purchased from Invitrogen (Darmstadt, Germany). U73122, U73343, 2-APB, BAPTA, CaCl_2_ and EGTA were obtained from Sigma-Aldrich (St. Louis, MO, USA). *Fluo-8* No Wash Calcium Assay Kit was purchased from Abcam (Cambridge, UK). All other chemicals were obtained from Roth (Karlsruhe, Germany).

### Cell culture and infection experiments

HBMEC were a kind gift from Prof. K.S. Kim [49]. HBMEC were regularly cultivated in RPMI-1640 medium (Biochrom AG, Berlin, Germany) containing 0.42 mM calcium, supplemented with 10% Fetal Calf Serum (FCS) (PAA Laboratories GmbH, Cölbe Germany), 10% Nu-Serum (BD Biosciences, Heidelberg, Germany), 2 mM L-glutamine, 1 mM sodium pyruvate, 1% minimal essential medium (MEM)-vitamins, and 1% non-essential amino acids (all from Gibco, Life Technologies, Karlsruhe, Germany), at 5% CO_2_, 37°C. Confluent HBMEC were diluted in fresh media and cultivated in tissue culture flasks maximum to passage 32. HBMEC were seeded on glass cover slips (diameter 12 mm) or directly in wells of a 24-well plate (Star lab, Hamburg, Germany). 2×10^5^ cells per well were cultured for 24 h prior to infection to form monolayers. Prior to the infection with meningococci, HBMEC were washed three times with Dulbecco's modified Eagle's medium (DMEM) without phenol red (Gibco, Life Technologies, Karlsruhe, Germany) supplemented with 1.0% FCS (infection medium). Endothelial cells were infected for four hours with *N. meningitidis* by using a multiplicity of infection (MOI) of 50 bacteria per host cell as described previously [43]. The infection dose (CFU) per cell/well was controlled by serial plating of meningococci on chocolate agar plates [43]. Infections were carried out for 4 h at 37°C and 5% CO_2_.

### Determination of free cytoplasmic calcium source in infected HBMEC

Several pharmacological inhibitors, which inhibit or modulate the calcium signaling pathway, were used in this study. Inhibitors were dissolved in dimethylsulfoxide (DMSO) or according to manufacturer instructions. HBMEC were pre-incubated with inhibitors at 37°C for 5–10 min prior to host cell infection, and the infection assays were performed in the presence of the inhibitors as described earlier [38]. Pharmacological inhibitors and DMSO had no effect on bacterial growth and on cell viability as determined by bacterial growth curve and trypan blue exclusion method after 4 h incubation. The trypan blue assay is based on the principle that dead cells take up certain dyes, such as trypan blue, Eosin and propidium iodide, whereas live cells having intact cell membranes, exclude such dyes. In this assay, cells are suspended with dye and examined under the fluorescence microscope to see the presence or absence of the dye in cytoplasm. Dead cells will take up the dye and will display blue stained cytoplasm, while viable cells will display a clear cytoplasm [50].

### Enumeration of adhered and internalized meningococci

To estimate the total amount of bacteria associated with host cells, unbound bacteria from the supernatant of the infected cells were removed by thoroughly washing with infection medium. Cells were lysed with saponin (1% w/v in DMEM) for 10 min, serial dilutions of lysate were plated on chocolate agar plates for over-night incubation at 37°C, 5% CO_2_ and total numbers of meningococci associated with endothelial cells (adhered and invasive bacteria) were quantified. Intracellular meningococci were determined after 1 h of incubation with infection medium containing gentamicin (Sigma-Aldrich St. Louis, MO, USA) at a concentration of 200 µg ml^−1^ and bacterial quantification was done as described above. The used antibiotic concentration killed all extracellular meningococci efficiently, however, no detrimental effect of the antibiotic was observed on internalized bacteria [43]. The adhered bacteria were estimated by subtracting the internalized bacteria from total associated bacteria. All experiments were performed in duplicate and repeated at least five times or as otherwise indicated.

### Measurement of intracellular calcium in HBMEC

Calcium imaging is a common and useful technique to measure calcium signaling in cultured cells. Calcium imaging techniques mostly take advantage of calcium indicator dyes that change their spectral properties in response to the binding of Ca^2+^ ions and act independent of dye concentration [51]. Cell permeable *Fluo*-8 no wash calcium assay kit (Abcam, Ltd., Cambridge, UK) was used to evaluate the effect of meningococci on intracellular calcium mobilization in HBMEC. Once *Fluo-8* is inside the cell, the lipophilic blocking groups of *Fluo-8* are cleaved by esterase, which results in a negatively charged fluorescent dye which remains inside the cells. Its fluorescence is greatly enhanced upon binding to calcium. The experiments were performed according to manufacturer instructions with minor modifications. Briefly, host cells were trypsinized and 8×10^4^ cells/well/100 µl were cultured overnight with regular growth medium in 96 well plates. Before calcium measurements growth medium was replaced with Hank’s balanced salt solution to minimize background fluorescence and compound interference with serum. Cells were treated with or without pharmacological inhibitors at indicated time points. Dye-loaded cells were further incubated for 30 min at 37°C and 5% CO_2_. Cytoplasmic calcium level at different time points was documented by monitoring the fluorescence intensity under a fluorescence microscope using a 20× objective lens. Alexa-Fluor-488 fluorescence channel with filters of appropriate wavelengths (Ex/Em = 490/565) was used to monitor the fluorescence intensity. The exposure time was kept constant during image acquisition of all samples. Meningococci at a MOI of 50 per cell were used. Bacteria were added to the well in presence or absence of inhibitors just before starting the fluorescence measurement. The change of free cytosolic calcium in HBMEC infected with meningococci was calculated as follows: the fluorescence intensity in HBMEC at different time points (Fx)/the fluorescence intensity in the same cells before infection (F0) x 100% [52], [53].

### Fluorescence staining and microscopy

Double immunofluorescence (DIF) staining and microscopy of infected host cells was carried out as described recently [54]. Briefly, the HBMEC grown on cover slips and infected with meningococci were washed repeatedly to remove unbound bacteria and fixed on glass cover slips (diameter, 12 mm) using 3.7% para-formaldehyde. Extracellular meningococci were incubated with a polyclonal anti-meningococcal antiserum [54] for 1 h and stained with secondary goat anti-chicken IgG coupled to Alexa-Fluor-594 (red) (Invitrogen Darmstadt, Germany). After washing, the host cells were permeabilized with 0.1% Triton X-100 for 10 min, and blocked with 3% BSA for 45 minutes. Extracellular and intracellular bacteria were incubated with anti-meningococcal antiserum for 1 h and later stained with secondary goat anti-chicken IgG coupled to Alexa-Fluor-488 (green) (Invitrogen, Darmstadt, Germany), while host cell nuclei were stained with DAPI. The coverslips were then mounted on microscope slides by using ProLongAntifadeReagent (Invitrogen, Darmstadt, Germany) and stored at 4°C in dark until examination. This staining procedure resulted in yellow (red and green) extracellular meningococci, green intracellular meningococci, and blue host cell nuclei. The stained samples were viewed with a Zeiss Apotome microscope using a 63×/1.4 objective lens. ZEN 2012 software was used for image acquisition (Carl Zeiss, Jena, Germany).

### Statistical analysis

All data are reported as mean ± standard deviation. Results were statistically analyzed using the paired two–tailed student’s t-test and a value of p<0.05 was accepted as indicating significance.

## Results

### *N. meningitidis* elevates cytoplasmic calcium in HBMEC

**Figure 1:**
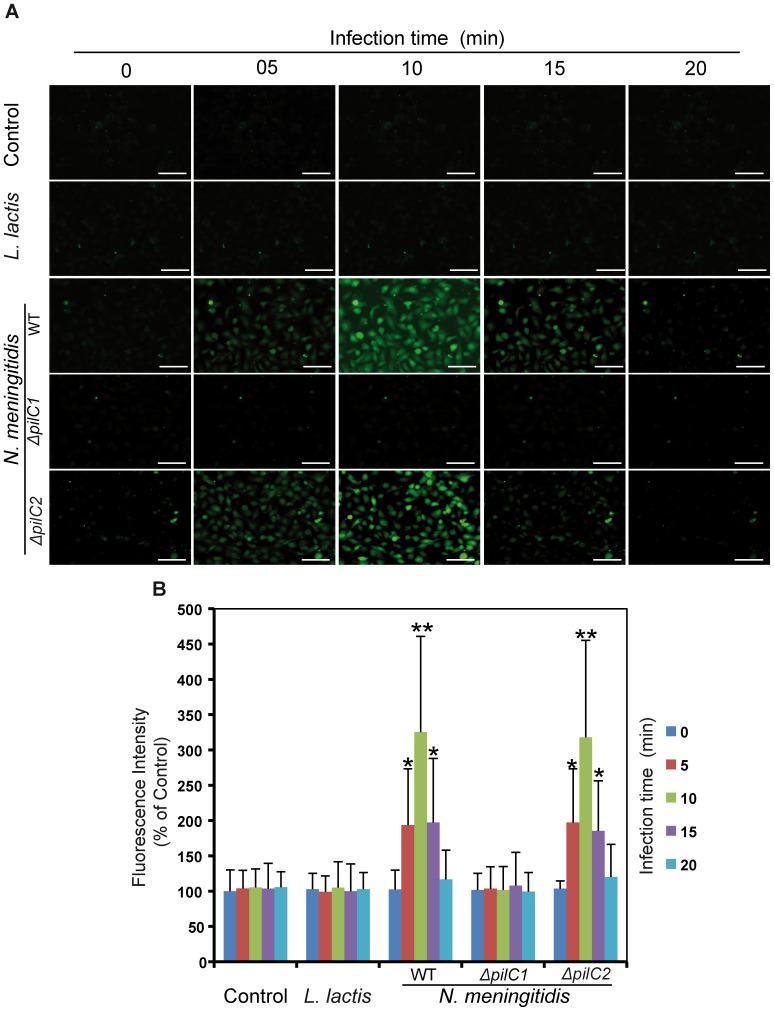
Meningococci mediate calcium mobilization in host cells via *pilC1.* (A) Changes of host cells intracellular calcium concentrations during infection with the meningococcal strains WT, *ΔpilC1* and *ΔpilC2* for the indicated times were monitored by performing immunofluorescence microscopy of *Fluo*-8 labeled HBMEC. Uninfected cells (control) and cells infected with *L. lactis* (*L. lactis*), respectively, were used as controls. Representative images from an individual experiment repeated five times in triplicate are shown. Scale bars represent 50 µm in all panels. (B) Quantification of changes in calcium concentrations in host endothelial cells during infection with different meningococcal strains are shown at the indicated time points. Uninfected cells (control) and cells infected with *L. lactis* (*L. lactis*), respectively, were used as controls. Data are expressed as the mean ± S.D of fifteen independent readings. *P<0.05, **P<0.01, versus values from control samples by Student’s *t*-test.

Cytosolic Ca^2+^ concentration was assessed by monitoring fluorescence intensity in HBMEC infected with meningococcal strains FAM20 (wild type), FAM20.1 (Δ*pilC1*), FAM20.2 (Δ*pilC2*) or infected with *L. lactis* or left uninfected. Meningococcal strains expressing *pilC1* (WT and Δ*pilC2*) significantly elevated the cytosolic Ca^2+^ concentration in infected cells compared to control or host cells infected with *L. lactis* (Figure 1A and 1B). However, Δ*pilC1* did not induce calcium efflux, and there was no difference in Ca^2+^ concentration compared to uninfected cells or host cells infected with *L. lactis* (Figure 1A and 1B). The transients in calcium concentration started after 5 min of bacterial infection, reached at peak within the next five minutes and almost went back to basal level after 20 min of infection (Figure 1). These results indicated that meningococcal *pilC1* caused host cell calcium concentration elevation.

### Interaction of PilC1 with HBMEC induces calcium release from intracellular stores

**Figure 2:**
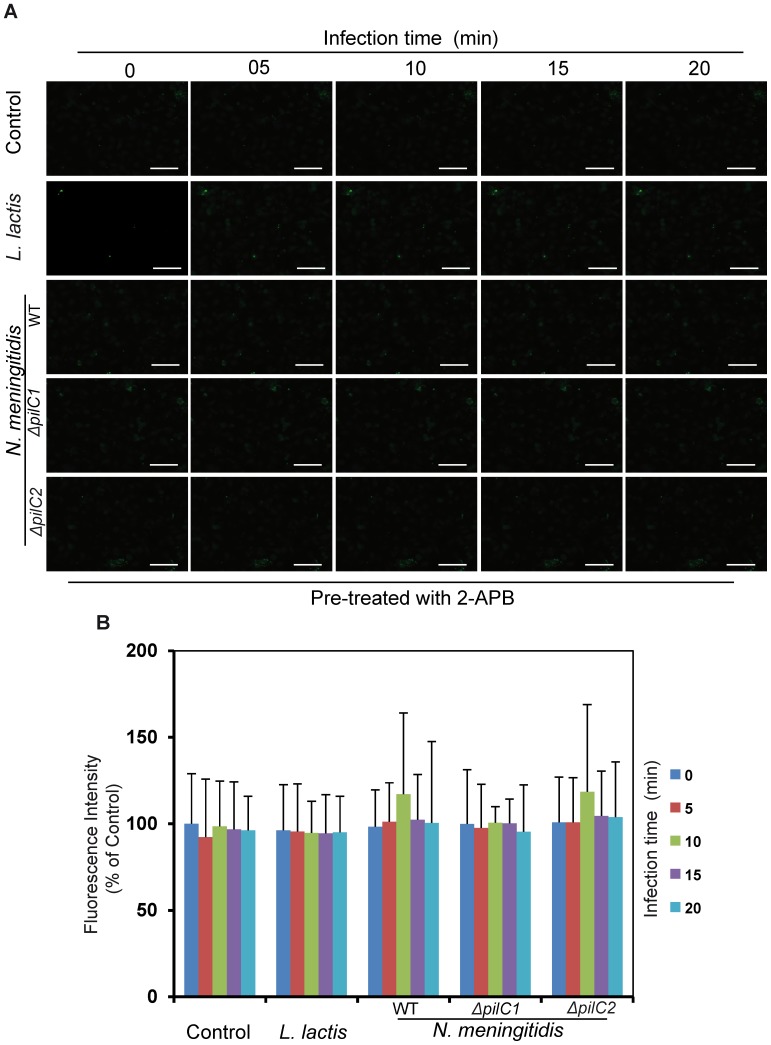
Meningococcal *pilC1* mediates calcium efflux from intracellular stores of host endothelial cells. HBMEC were pre-treated with 50 µM of 2-APB before meningococcal infection. 2-APB inhibits IP_3_ receptors and blocks release of calcium from store operated channels. Fluorescence microscopy was performed at the indicated time points. Uninfected cells (control) and cells infected with *L. lactis* (*L. lactis*), respectively, were used as controls. Representative images from an individual experiment repeated five times in triplicate are shown. Scale bars represent 50 µm in all panels. (B) Quantification of changes in calcium concentrations in host endothelial cells pre-treated with 2-APB during infection with different meningococcal strains are shown at the indicated time points. Uninfected cells (control) and cells infected with *L. lactis* (*L. lactis*), respectively, were used as controls. Data are expressed as the mean ± S.D of fifteen independent readings. *P<0.05, **P<0.01, versus values from control samples by Student’s *t*-test.

To further determine if cytosolic Ca^2+^ changes in HBMEC observed during meningococcal infection were due to influx of calcium from the extracellular medium or release from intracellular stores, we used 2-aminobiphenyl borate (2-APB), a cell-permeable channel blocker of inositol 1,4,5-triphosphate (InsP_3_) receptors on endoplasmic reticulum [55]. The endoplasmic reticulum (ER) is the main organelle capable of taking up, storing and releasing calcium ions, serves as a dynamic Ca^2+^ pool in almost all eukaryotic cells and is involved in Ca^2+^ signaling upon stimulation [56], [57]. InsP_3_ serves as a channel for Ca^2+^ release from intracellular Ca^2+^ stores (ER) upon stimulation [57] and can be efficiently blocked by 2-APB [55]. Therefore, HBMEC were treated with 2-APB (50 µM) prior to infection with meningococci, and Ca^2+^ concentrations were monitored by immunofluorescence microscopy (Figure 2A and 2B). Interestingly, calcium efflux induced by meningococcal PilC1 was abolished under the effect of 2-APB (Figure 2A and 2B), suggesting that Ca^2+^ release in response to PilC1- expressing meningococci (WT and *ΔpilC2*) was from intracellular stores of HBMEC (Figure 1 and 2).

#### Cytoplasmic Ca^2+^ concentrations play a critical role during adherence and invasion of *N. meningitidis* to host cells

**Figure 3:**
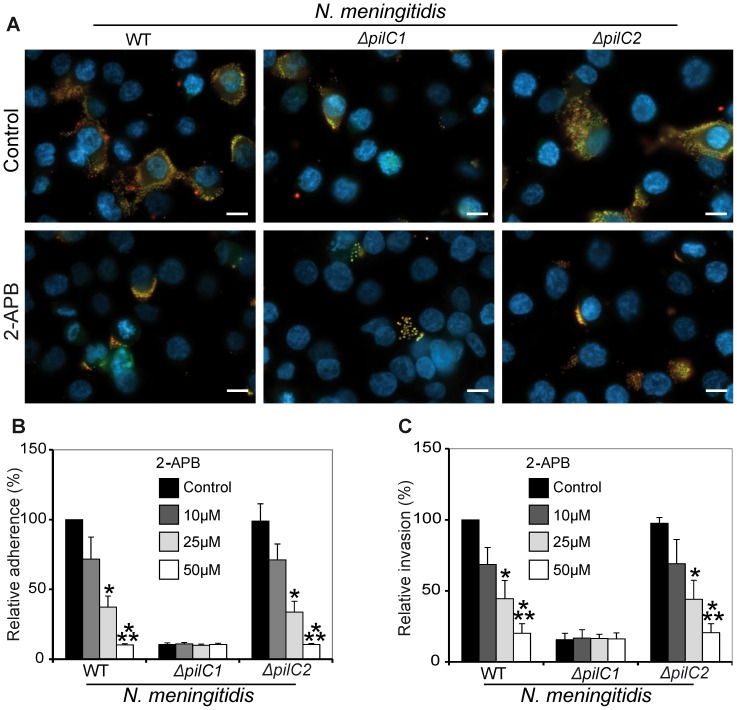
Intracellular release of calcium ions facilitates meningococcal internalization. HBMEC were grown on (A) cover slips or (B and C) directly in wells of 24 well plates. Host cells were treated with or without 2-APB and infected with meningococcal strains WT, *ΔpilC1* and *ΔpilC2* for 4 h at 37°C. (A) After removing unbound bacteria by washing, adherent meningococci were incubated with chicken anti-meningococcal primary IgG and Alexa-Fluor-594 (red) coupled anti-chicken secondary antibodies. Cells were permeabilized by Triton X-100 and extra- and intracellular meningococci were further incubated with chicken anti-meningococcal IgG and Alexa-Fluor-488 (green) coupled anti-chicken antibodies. DAPI was used to stain the host cell nuclei. Fluorescence microscopy was performed using a Zeiss Axiobserver Z1 microscope equipped with an Apotome. Extracellular bacteria appear yellow (red and green) and intracellular bacteria appear red. Representative images from an individual experiment repeated five times in triplicate are shown. Scale bars represent 50 µm in all panels. (B) The total number of bacteria associated with host cells was determined after removing unbound extracellular bacteria by washing the cells grown in wells of 24 well plates with infection medium. Host cells were lysed with saponin followed by plating of the serially diluted cells on chocolate agar plates overnight at 37°C. Control of WT was taken as 100% and other values are presented as relative to this control. (C) After removing unbound bacteria by repeated washing steps, the number of internalized meningococci was determined by the antibiotic protection assay after 4 h of infection in presence of 2-APB. Data are expressed as the mean ± S.D of five independent experiments performed in duplicate. *P<0.05, ***P<0.001, versus values from control samples by Student’s *t*-test.

To investigate the role of free cytosolic Ca^2+^ concentrations during the internalization process of meningococci into HBMEC, host cells were pre-treated with 2-APB (50 µM) for 30 min to block calcium signaling *via* IP3 receptors on the endoplasmic reticulum surface. Total associated meningococci were estimated by performing DIF staining (Figure 3A) as has been described in Material and Methods. The pre-treatment of 2-APB causes a significant decrease of bacterial host cell association in case of *pilC1*- expressing meningococci relative to respective controls (Figure 3A). However, blocking of calcium signaling has no effect on bacterial association to host cells in case of *ΔpilC1* (middle panel Figure 3A). The results demonstrate that calcium signaling is required for efficient adherence and internalization of meningococci into cells of the BBB. To further confirm these results, data were quantified by performing gentamicin protection assays as described in Material and Methods. The quantified data further confirmed the pattern observed by immunofluorescence staining. Presence of 2-APB decreased the number of adhered bacteria from 1.35×10^6^ and 1.5×10^6^ to 1.31×10^5^ and 1.36×10^6^ for WT and *ΔpilC2*, respectively. About 80% decrease in adherence (Figure 3B) and subsequent invasion of PilC1-expressing meningococci was observed due to pre-treatment of host cells with 2-APB (Figure 3B and 3C). However, under the effect of 2-APB no difference in adherence and invasion of the PilC1-deficient meningococcal strain (*ΔpilC1*) was observed (Figure 3B and 3C). The numbers of adhered *ΔpilC1* meningococci in absence or presence of 2-APB remained 1.26×10^5^ and 1.27×10^5^, respectively. This depicts no inhibitory effect of 2-APB on *ΔpilC1*. Taken together, these data suggest a critical role of cytoplasmic calcium concentrations during PilC1-mediated meningococcal adherence to and invasion into host endothelial cells.

#### Role of Phospholipase C during meningococcal interaction with HBMEC

**Figure 4:**
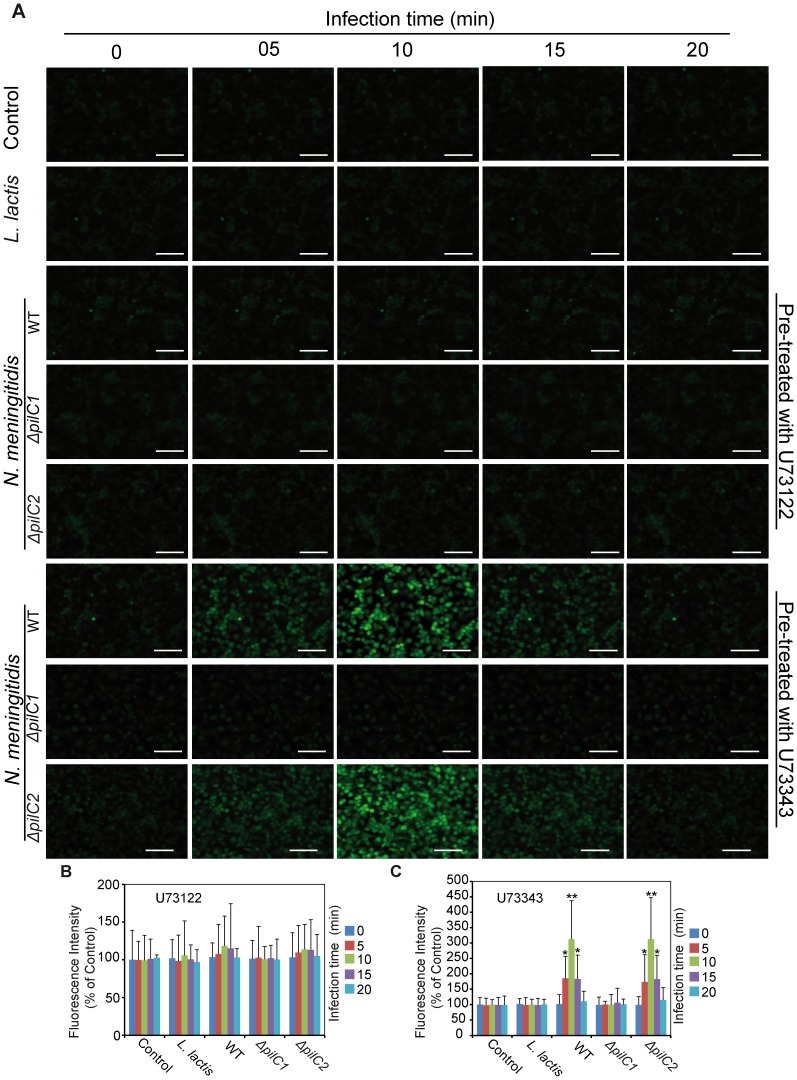
Phospholipase C is required to induce calcium signaling by meningococci. Host endothelial cells were treated with *Fluo*-8 calcium dye for 1 h prior to infection with meningococcal strains. (A, B and C) Cells were either treated with the PLC inhibitor U73122 or its negative control U73343. Uninfected cells (control) and cells infected with *L. lactis* (*L. lactis*), respectively, were used as controls to monitor the calcium fluxes by fluorescence microscope. (A) Representative images from an individual experiment repeated five times in triplicate are shown. U73122 significantly blocked the calcium efflux mediated by meningococci (*pilC1^+^* strains) compared to non-treated or control-treated (U73343) cells. Scale bars represent 50 µm in all panels. (B and C) Data are expressed as the mean ± S.D of five independent experiments performed in duplicate. *P<0.05, ***P<0.001, versus values from control samples by Student’s *t*-test.

Cytosolic Ca^2+^ is regulated by several different pathways [57]. Among these pathways, the PLC signaling pathway has also been reported to play a critical role in mediating Ca^2+^ signaling in host cells during infection processes of different pathogens by catalyzing phosphatidylinositol-4,5-biphosphate (PIP2) to form inositol-1,4,5-triphosphate (IP3) [38], [58], [59]. To determine the role of PLC during meningococcal infection, HBMEC were either pre-treated with the cell permeable PLC inhibitor U73122 or its inactive analogue U73343 [59] or left untreated as control. U73122 is a specific inhibitor of phospholipase C and thereby inhibits the generation of InsP_3_ and the release of Ca^2+^ from intracellular stores in various cell types, whereas its inactive analogue U73343 has no effect and was employed as a negative control [59]. Cells infected with *L. lactis* were also used as control (Figure 4A and 4B). Results indicated that pre-treatment of host cells with U73122 significantly blocked meningococcal PilC1-mediated Ca^2+^ release in HBMEC compared to cells treated with the inactive analogue U73343. Taken together, these data suggest that activation of PLC plays a critical role during PilC1- mediated meningococcal calcium release in host endothelial cells.

**Figure 5:**
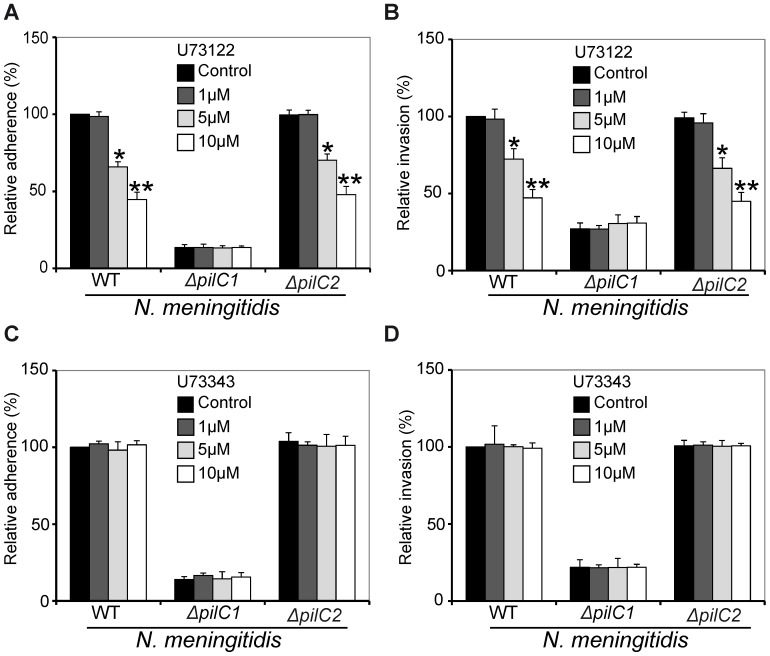
Meningococcal *pilC1-*mediated adherence to and invasion into host cells require PLC. Host cells were grown in wells of 24 well plates, pre-treated with different concentrations of U73122 (A and B), U73343 (C and D) or left untreated (control). Infection assays with three meningococcal strains (WT, *ΔpilC1* and *ΔpilC2*) were carried out for 4 h at 37°C and 5% CO_2_. (A and C) The total number of bacteria associated with host cells was determined after removing unbound bacteria by washing the cells grown in wells of 24 well plates with infection medium. Subsequently, host cells were lysed with saponin and serial dilutions of the lysates were plated on chocolate agar and incubated overnight at 37°C. Control of WT was taken as 100% and other values are presented as relative to this control. (B and D) After removing unbound bacteria by washing, the number of internalized meningococci was determined by the antibiotic protection assay after 4 h of infection in presence of 2-APB. Data are expressed as the mean ± S.D of five independent experiments performed in duplicate. *P<0.05, **P<0.01 versus values from control samples by Student’s *t*-test.

To further decipher the role of PLC during adherence and subsequent invasion of meningococci in HBMEC, U73122 and U73343 were used during infection assay. HBMEC pre-treated with U73122, U73343 or controls were infected with three different meningococcal strains for the indicated time and concentrations of the inhibitors (Figure 5). Enumeration of adhered and internalized meningococcal enumeration was performed as described in Material and Methods. The data revealed a significant decrease in PilC1 expressing meningococcal (WT and *ΔpilC2*) adherence to and invasion into host cells under the effect of U73122 (Figure 5A and 5B). However, no effect of U73122 was observed on adherence and invasion of *ΔpilC1* (Figure 5A and 5B). Almost 50% decrease in meningococcal (WT and *ΔpilC2*) adherence (Figure 5A) and invasion (Figure 5B) to HBMEC pre-treated with U73122 was observed. As expected, U73343, the inactive analogue of U73122, demonstrated no significant effect on all used meningococcal strains (Figure 5C and 5D). Immunofluorescence staining also confirmed the above mentioned observations (1). These results deciphered the key role of PLC during meningococcal interaction with the BBB.

#### Chelation of free cytosolic Ca^2+^ has an inhibitory effect on meningococcal interaction to HBMEC

**Figure 6:**
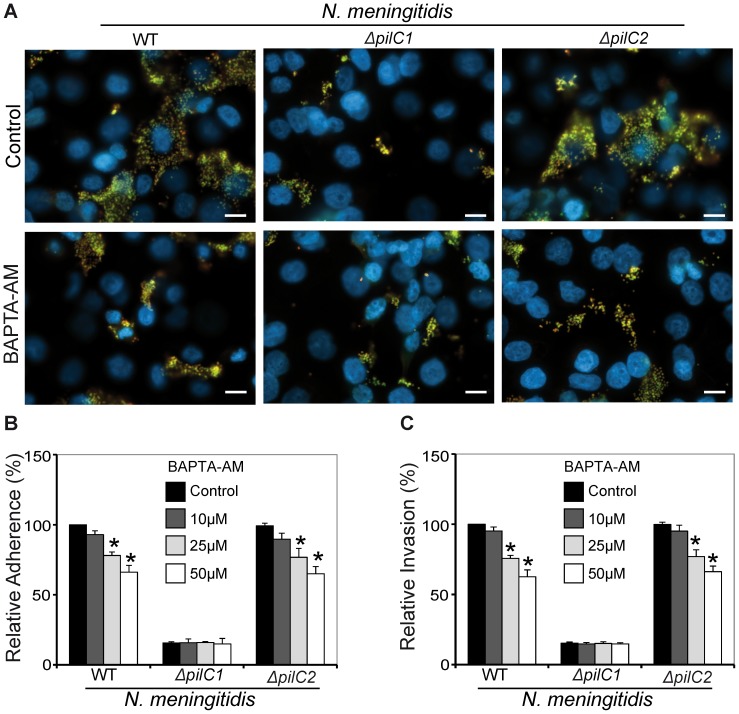
Chelating intracellular calcium inhibits meningococcal invasion into host endothelial cells. BAPTA-AM (50 µM) pre-treated host cells were grown on (A) glass cover slips for microscopy or directly in (B and C) wells of 24 well plates to quantify the adhered and internalized bacteria. HBMEC were infected with meningococci (MOI50) for 4 h at 37°C and 5% CO_2_. After infection, host cells were washed thoroughly to remove the unbound meningococci. (A) Cells were fixed with paraformaldehyde, incubated with anti-meningococcal IGg, followed by an Alexa-Fluor-594 labelled secondary antibody. Host cells were permeabilized to stain intracellular bacteria with Alexa-Fluor-488 and nuclei with DAPI. Scale bars represent 50 µm. (B) The number of adhered meningococci to host cells grown in wells of 24 well plates was determined after removing unbound extracellular bacteria and plating the recovered bacteria on blood agar plates. (C) Effect of pre-treating host endothelial cells with BAPTA-AM on internalization meningococci after 4 h of infection of HBMEC as determined by the antibiotic protection assay. Adherence and internalization of WT meningococci by host cells in the absence of BAPTA-AM was set to 100% and other values are presented as relative percentage relative to the WT control. Data represent means ± S.D (n = 5) (*P<0.05).

To examine the role of intracellular calcium concentrations on meningococcal adherence and subsequent invasion, we used BAPTA-AM in infection experiments. BAPTA-AM is a cell permeable cytosolic calcium chelator [60]. Treatment of eukaryotic cells with this pharmacological inhibitor results in lower cytosolic calcium concentration due to closure of calcium release channels located on the endoplasmic reticulum surface [61]. Thus, HBMEC were treated with BAPTA-AM for 30 min prior to infection with meningococci. Adherence and invasion were estimated by performing DIF staining or the bacterial colony forming units were determined by plating the bacteria on chocolate agar plates for overnight at 37°C and 5% CO_2_. Interestingly, the results demonstrated a significant decrease in adherence and invasion of PilC1-expressing meningococci to HBMEC (Figure 6A, 6B and 6C). However, adherence and invasion of the PilC1*-*deficient meningococcal strain (*ΔpilC1*) remained unchanged (Figure 6A, 6B and 6C). DIF staining revealed that the numbers of meningococci (WT and *ΔpilC2*) associated with host cells were strongly reduced under the effect of BAPTA-AM (Figure 6A). Furthermore, enumeration of adhered and internalized bacteria showed a significant decrease due to intracellular calcium chelation by BAPTA-AM (Figure 6B and 6C). This inhibitory effect was observed in a dose-dependent manner (Figure 6B and 6C). Importantly, the effect of BAPTA-AM was only observed in case of PilC1-expressing meningococci and the interaction of the PilC1*-*deficient meningococcal strain (*ΔpilC1*) with host cells remained unchanged (Figure 6). Moreover, the effect of intracellular calcium chelation subsequently to bacterial infection was monitored by administering BAPTA-AM, 20 min post bacterial infection to host cells (Fig. S2). As expected, there was no significant effect on meningococcal association to host cells. Taken together, these results indicated that manipulation of calcium signaling pathways and buffering of intracellular calcium by pharmacological inhibitors prior to bacterial infection resulted in impaired meningococcal adherence to and invasion into HBMEC.

#### Extracellular Ca^2+^ concentration has no significant effect on meningococcal adherence and invasion to HBMEC

**Figure 7:**
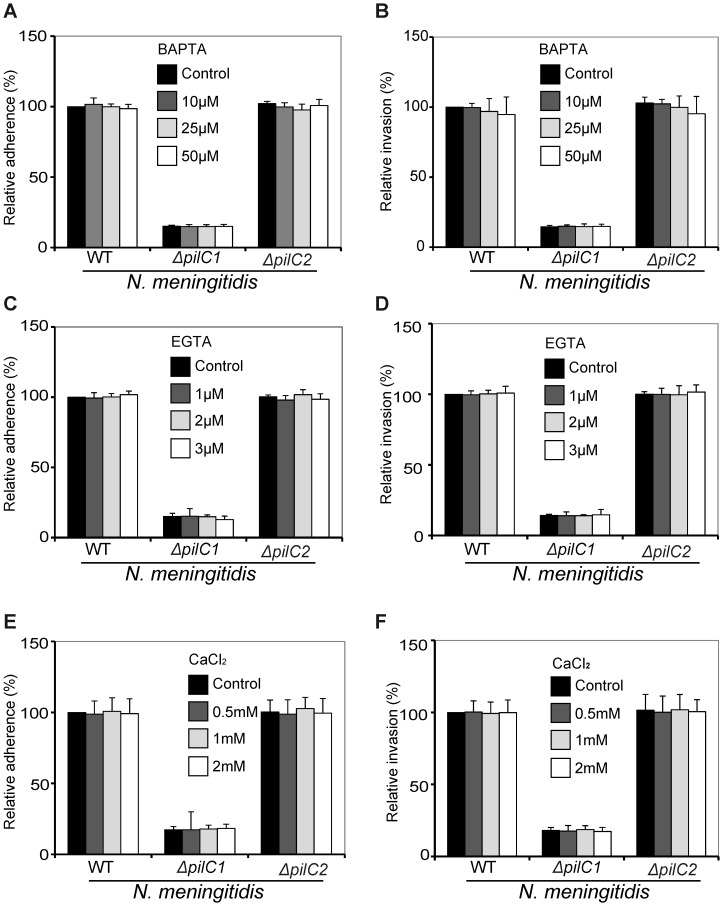
Extracellular Ca^2+^ concentration has no effect on meningococcal adherence to and subsequent invasion into HBMEC. Host cells pre-treated with extracellular calcium chelators BAPTA and EGTA or CaCl_2_ were infected with three different strains of meningococci with MOI of 50 for 4 h at 37°C and 5% CO_2_. (A, C and E) The number of meningococci adhered to host endothelial cells was determined in the absence (control) or presence of BAPTA, EGTA or CaCl_2_ by lysing the host cells and plating of the lysates on agar plates for overnight at 37°C and 5% CO_2_. (B, D and F) The number of meningococci invaded into HBMEC was determined by using the antibiotic protection assay. Adherence and invasion of WT meningococci in the absence of inhibitors was set to 100%. Meningococcal adherence to and subsequent invasion into host cells was not altered by manipulation of the extracellular calcium. Data represent means ± S.D (n = 5) (*P<0.01).

In order to determine if extracellular calcium concentrations influence meningococcal association and entry to host cells, HBMEC were either pre-treated with the extracellular calcium chelators BAPTA (non-cell permeable) and EGTA or CaCl_2_ was added to increase the calcium level in the extracellular medium. Infection assays were carried out as described above. The effect of low or high extracellular calcium concentration on meningococcal adherence and invasion was investigated separately by plating the associated and internalized bacteria on chocolate agar plates overnight at 37°C and 5% CO_2_. The results showed that extracellular calcium has no significant effect on meningococcal adherence to (Figure 7A, 7C and 7E) and invasion (Figure 7B, 7D and 7F) into host endothelial cells. Double immunofluorescence staining also confirmed that employing BAPTA, EGTA or CaCl_2_ did not affect the meningococcal association to and internalization into host endothelial cells (Figure S3). Taken together, this study demonstrates that PilC1 of meningococci mediates calcium efflux from intracellular calcium stores of HBMEC via a PLC pathway. This calcium mobilization plays a key role during meningococcal adherence to and subsequent invasion into host cells.

## Discussion

Meningococci employ several virulence factors including surface-expressed proteins such as Opa, Opc and type IV pili to invade host cells. Type IV pili are crucial for bacterial adhesion to host cells as non-piliated encapsulated meningococci are unable to interact with human cells [8]. These pili facilitate meningococcal division of initially attached bacteria on host cell surfaces and thus increase the number of bacteria interacting with host cells [2]. PilE encodes the major subunit of meningococcal pili and is involved in antigenic variation, which modulates the degree of bacterial virulence. PilC is known as a minor subunit of meningococcal pili and carries two homologous alleles, *pilC1* and *pilC2*, which are expressed independently of each other. Expression of the PilC1 protein has been shown to be critical for meningococcal adherence to host cells [15].

Ca^2*+*^ plays a key role as a second messenger to regulate several cellular functions such as gene expression, vascular contraction, cytoskeletal rearrangements and cell signaling [30]. Interestingly, several bacterial pathogens have evolved strategies to interfere with intracellular calcium signaling in order to promote their invasion into host cells [31], [33], [38]. However, the impact of calcium signaling and cytoplasmic concentrations of calcium in host cells on meningococcal adherence to and invasion into human endothelial cells are the major questions yet to be answered.

This study deciphers the role of calcium signaling mediated by PilC1 during meningococcal adherence and subsequent invasion of host endothelial cells in detail. Meningococcal strains expressing (FAM20 and FAM20.2) or lacking PilC1 (FAM20.1) were used to analyze the interaction with HBMEC, which are commonly used as *in vitro* model of the human BBB [49]. Several pathogens employ their virulence factor(s) to mediate calcium signaling in host cells [31], [33], [38], [58], meningococcal PilC1 serves the same purpose here. This hypothesis was confirmed as a meningococcal strain deficient of PilC1 (FAM20.1) was not able to increase the cytosolic Ca^2*+*^ concentration in host cells. The fluorescence microscopy of host cells loaded with the calcium-binding fluorescence dye *Fluo-8* and infected with meningococci showed significantly increased fluorescence intensity (increase in cytosolic calcium) after 10 min of bacterial addition. Free cytosolic calcium in host cells was observed for a minimum of 20 min post infection to fully monitor the signaling cascade. To rule out the effect of non-specific calcium signaling due to bacterial stimulation, *L. lactis* were used as a control along with uninfected cells. However, PilC1-expressing meningococci exhibited significantly higher cytoplasmic calcium concentrations in HBMEC. Similar effects caused by virulence factors of *S. pneumonia, Chlamydia trachomatis* (*C. trachomatis*) and *C. Jejuni* have been reported previously [35], [38], [62]. Virulence factors of *S. pneumonia* (PspC) *and C. trachomatis* (elementary bodies) induce Ca^2+^ influx, which in turn facilitates bacterial internalization to host cells.

In response to external stimuli Ca^2+^ transients in eukaryotic cells mainly occur either via influx of exogenous Ca^2+^ into the cell or release of Ca^2+^ from internal stores as the endoplasmic reticulum (ER). The classical pathway to release Ca^2+^ ions from intracellular stores (i.e. ER) is the stimulation of muscarinic receptors to activate PLC. PLC catalyzes PIP2 to IP3, which in turn stimulates the receptors located on calcium stores to release calcium [55], [63]. To dissect between extracellular calcium influx and release of calcium from intracellular stores in host cells, specific inhibitors have been employed in this study as has been described previously [55].

2-APB acts as an IP3-specific receptor antagonist and inhibits Ca^2+^ release via store-operated calcium channels (SOCs) [55], [64]. In presence of 2-APB, calcium efflux induced by PilC1-expressing meningococci (WT and *ΔpilC2*) was almost completely blocked. Additionally, meningococcal adherence and subsequent invasion was significantly impaired in 2-APB pre-treated host cells as observed by DIF staining and gentamicin protection assays. These results indicate a key role of calcium release from intracellular stores via IP3 receptors during meningococcal interaction with host endothelial cells. These findings are in agreement with previous studies performed with different bacterial pathogens [31], [32], [35], [38], [65]. For example, C. *jejuni* induces calcium transients via IP3 receptors during interaction with host epithelial cells [35] and these effects were significantly abolished in presence of 2-APB.

Since, PLC catalyzes the conversion of PIP2 to IP3, which leads to release of calcium from intracellular stores [66], the activation of PLC is required for formation of IP3 and calcium efflux from intracellular stores. The pharmacological substance U73122 is commonly used as an inhibitor of PLC. Efficient internalization of several pathogens to their respective host cells has been linked to PLC activity [35], [38], [67]. For example, pre-treatment of HBMEC with a PLC inhibitor (U73122) almost completely blocked the traversal of *Borrelia burgdorferi* (*B. burgdorferi*) [67]. Similarly, efficient internalization of *C. jejuni* into human embryonic intestinal cells (INT407) is also dependent on activation of PLC [35]. This study shows that PLC plays a critical role and regulates the calcium signaling during meningococcal interaction with host endothelial cells. Cells pre-treated with a PLC inhibitor (U73122) were unable to release calcium from intracellular stores in response to meningococci expressing PilC1 (WT and *ΔpilC2*). However, release of calcium was not effected in cells pre-treated with U73343 (inactive analogue of U73122) or untreated cells (control). Furthermore, inactivation of PLC significantly reduced the adherence to and invasion into host endothelial cells. These results clearly indicate that meningococci induced the release of calcium from endoplasmic reticulum via a PLC-regulated pathway and dependent on the expression of PilC1 protein by meningococci.

To gain effective invasion into host cells, many pathogens subvert the host cell signaling system, which may result in phosphorylation of certain host cell proteins and rearrangements of the host cytoskeleton. For example, *S. typhimurium* and enteropathogenic *E. coli* (EPEC) infection of host cells induce calcium release by activating IP3 receptors on cellular stores [31], [32], [65]. Calcium transients induced by EPEC were monitored by immunofluorescence microscopy, and only virulent strains were able to cause calcium transients in HEp2 cells [65]. Studies have also revealed that actin pedestal formation by enteropathogenic *E. coli* is regulated by calcium signaling along with other effector proteins [68]. Invasive strains of *S. typhimurium* have been reported to induce rapid increase in the levels of free intracellular calcium and mutants defective in invasion were unable to induce these calcium fluxes. Furthermore, addition of calcium antagonists blocked the wild-type *S. typhimurium* invasion into host cells [31]. Similar to our findings, PilY1 of *P. aeruginosa* binds to integrins in a calcium-dependent manner and is also required for bacterial twitching and swarming motility as well as adherence to target host cells [36], [69]. The pilus-associated protein PilY1 of *P. aeruginosa* shares functional similarities with neisserial proteins. Studies have demonstrated that either calcium chelation or mutation of a single residue in PilY1 blocked the bacterial twitching motility and binding to host integrin, which resulted in low adherence to host cells [36], [69]. Furthermore, it has been shown that PilC1 of *K. kingae* and *N. gonorrhoeae* shares homology with the calcium-binding site of PilY1 from *P. aeruginosa* [37], [70]. Studies performed with PilC1 and PilC2 with mutations in their calcium-binding domains demonstrated that only PilC is necessary for adherence to host epithelial cells, whereas PilC1 and PilC2 are dispensable for bacterial piliation [37], [70]. Importantly, our findings are in agreement with these studies.

An increased cytosolic calcium concentration has been reported to play an important role in virulence of bacterial pathogens. For example, *S. pneumoniae* induces calcium efflux in host epithelial cells via the interaction of the pneumococcal surface protein C (PspC) with human polymeric immunoglobulin receptor (hpIgR), and release of calcium from intracellular stores promote bacterial internalization to Calu-3 and MDCK-hpIgR epithelial cells [38]. Moreover, heterologous expression of the *S. pneumoniae* virulence factor PspC on the surface of *L. lactis* also induced calcium release from intracellular stores and calcium signaling enhanced bacterial adherence and subsequent invasion into host epithelial cells. Similar to our findings, intracellular Ca^2+^ concentration has been associated with the invasion of *C. jejuni, L. monocytogenes,* and *C. trachomatis* [33]-[35], [62]. *C. trachomatis* has been reported to mobilize Ca^2+^ in intracellular stores of host cells via its elementary bodies, which promotes internalization and intracellular survival of this pathogen [62]. Invasion of *C. jejuni* is dependent on calcium transients and markedly reduced either by chelating host intracellular Ca^2+^ with BAPTA-AM or by blocking the release of Ca^2+^ from intracellular stores with U73122, however, buffering of extracellular calcium with EGTA or BAPTA has no effect on bacterial invasion into host cells [35]. These findings are in agreement with our results and importantly such calcium fluxes are required at early time points of meningococcal interaction with host cells to trigger the signaling cascades, which in turn promote their adherence to host cells. Once this signaling plethora has been switched on it cannot be blocked or reversed by employing the calcium chelating inhibitors, as has been demonstrated by addition of BAPTA-AM at 20 min post meningococcal infection.

In conclusion, this study demonstrates clearly that meningococci mediate Ca^2*+*^ release in host endothelial cells via PilC1 in a PLC-dependent manner by activating the IP3 receptors on the surface of the endoplasmic reticulum. Furthermore, PilC1-mediated meningococcal adherence to and invasion into HBMEC are associated with host intracellular calcium content but are independent of extracellular calcium concentrations. Buffering the cytosolic calcium resulted in a higher number of meningococci adhered to host cells and facilitate meningococcal internalization into endothelial cells of the BBB.

## Supporting Information

Figure S1 **Role of PLC during** ***pilC1*** **mediated calcium signaling in host cells.** HBMEC were grown on cover slips, pre-treated with U73122 (10 µM), U73343 (10 µM) or left untreated (control). Infection assays were carried out with three different meningococcal strains (WT, *ΔpilC1* and *ΔpilC2*) for 4 h at 37°C and 5% CO_2_. DIF and fluorescence microscopy were performed as described in Materials and Methods.
(TIF)

Figure S2 **Intracellular calcium chelation after bacterial infection has no effect on meningococcal interaction with host cells.** Host cells grown on (A) glass cover slips for microscopy or directly in (B and C) wells of 24 well plates to quantify the adhered and internalized bacteria. Host cells were infected with the indicated meningococcal strains for 20 min and subsequently treated with BAPTA-AM. HBMEC were infected with meningococci (MOI50) for 4 h at 37°C and 5% CO_2_. After infection, host cells were washed thoroughly to remove the unbound meningococci. (A) Cells were fixed with paraformaldehyde, incubated with anti-meningococcal IGg, followed by an Alexa-Fluor-594 labelled secondary antibody. Host cells were permeabilized to stain intracellular bacteria with Alexa-Fluor-488 and nuclei with DAPI. Scale bars represent 50 µm. (B) The number of adhered meningococci to host cells grown in wells of 24 well plates was determined after removing unbound extracellular bacteria and plating the recovered bacteria on blood agar plates. (C) Effect of BAPTA-AM (20 min post infection) on meningococcal internalization to cells as determined by the antibiotic protection assay. Adherence and internalization of WT meningococci by host cells in the absence of BAPTA-AM was set to 100% and other values are presented as relative percentage of the WT control. Data represent means ± S.D (n = 5) (*P<0.05).
(TIF)

Figure S3 **Extracellular Ca^2+^ concentration has no effect on meningococcal adherence to and subsequent invasion into HBMEC**. Host cells pre-treated with extracellular calcium chelators BAPTA and EGTA were infected with three different strains of meningococci (WT, *ΔpilC1* and *ΔpilC2*) with an MOI of 50 for 4 h at 37°C and 5% CO_2_. Adhered and internalized meningococci to host endothelial cells were visualized by double immunofluorescence staining and fluorescence microscopy as described in Materials and Methods.
(TIF)
