# Supplementary figures and images for: Impact of Calcium Signaling during Infection of Neisseria meningitidis to Human Brain Microvascular Endothelial Cells
Source: PLoS One. 2014 Dec 2;9(12):e114474. doi: 10.1371/journal.pone.0114474 (PMC4252121; doi:10.1371/journal.pone.0114474)

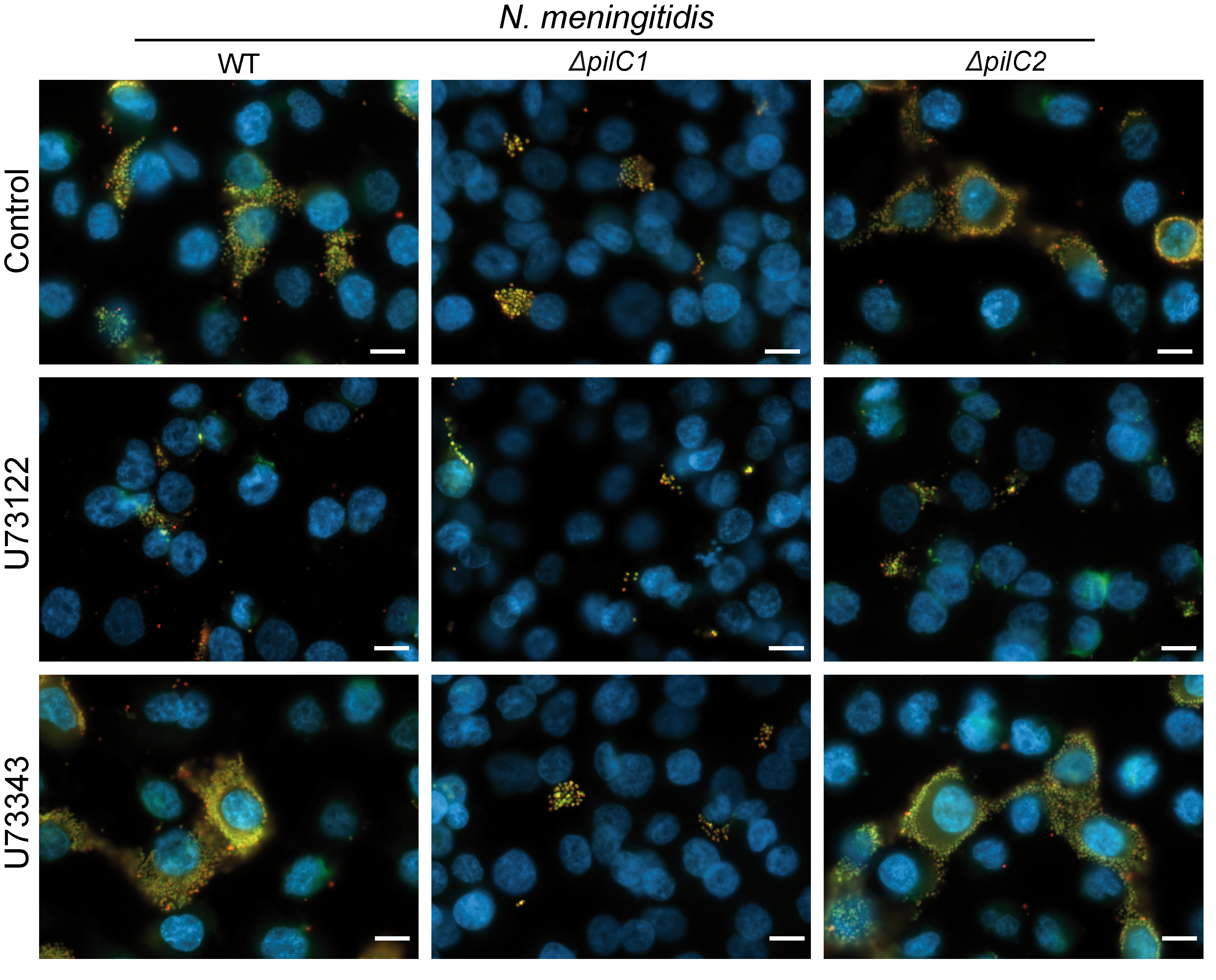

Supplement: Figure S1 — Role of PLC during pilC1 mediated calcium signaling in host cells. HBMEC were grown on cover slips, pre-treated with U73122 (10 µM), U73343 (10 µM) or left untreated (control). Infection assays were carried out with three different meningococcal strains (WT, ΔpilC1 and ΔpilC2) for 4 h at 37°C and 5% CO2. DIF and fluorescence microscopy were performed as described in Materials and Methods. (TIF) [file pone.0114474.s001.tif]

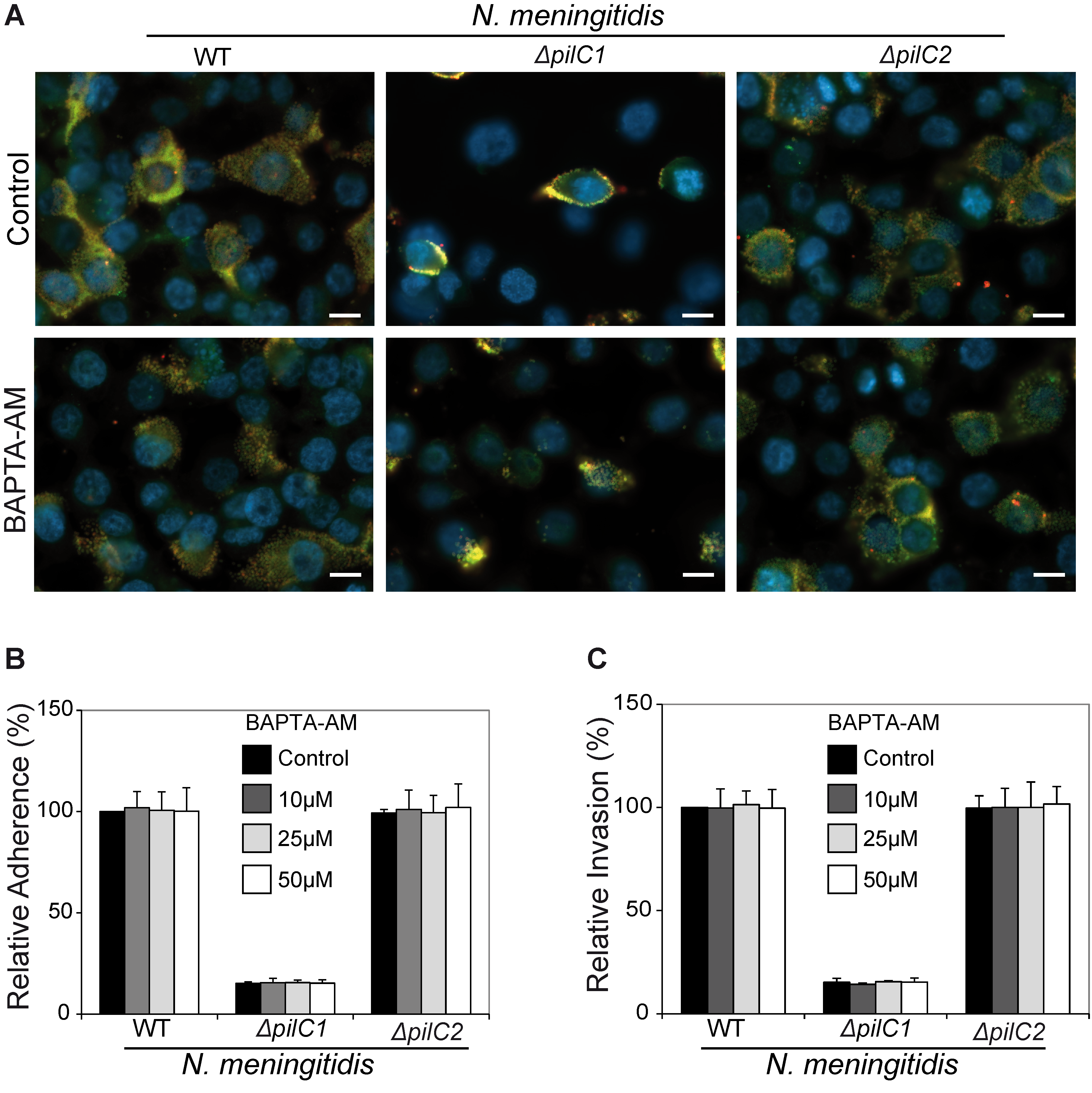

Supplement: Figure S2 — Intracellular calcium chelation after bacterial infection has no effect on meningococcal interaction with host cells. Host cells grown on (A) glass cover slips for microscopy or directly in (B and C) wells of 24 well plates to quantify the adhered and internalized bacteria. Host cells were infected with the indicated meningococcal strains for 20 min and subsequently treated with BAPTA-AM. HBMEC were infected with meningococci (MOI50) for 4 h at 37°C and 5% CO2. After infection, host cells were washed thoroughly to remove the unbound meningococci. (A) Cells were fixed with paraformaldehyde, incubated with anti-meningococcal IGg, followed by an Alexa-Fluor-594 labelled secondary antibody. Host cells were permeabilized to stain intracellular bacteria with Alexa-Fluor-488 and nuclei with DAPI. Scale bars represent 50 µm. (B) The number of adhered meningococci to host cells grown in wells of 24 well plates was determined after removing unbound extracellular bacteria and plating the recovered bacteria on blood agar plates. (C) Effect of BAPTA-AM (20 min post infection) on meningococcal internalization to cells as determined by the antibiotic protection assay. Adherence and internalization of WT meningococci by host cells in the absence of BAPTA-AM was set to 100% and other values are presented as relative percentage of the WT control. Data represent means ± S.D (n = 5) (*P<0.05). (TIF) [file pone.0114474.s002.tif]

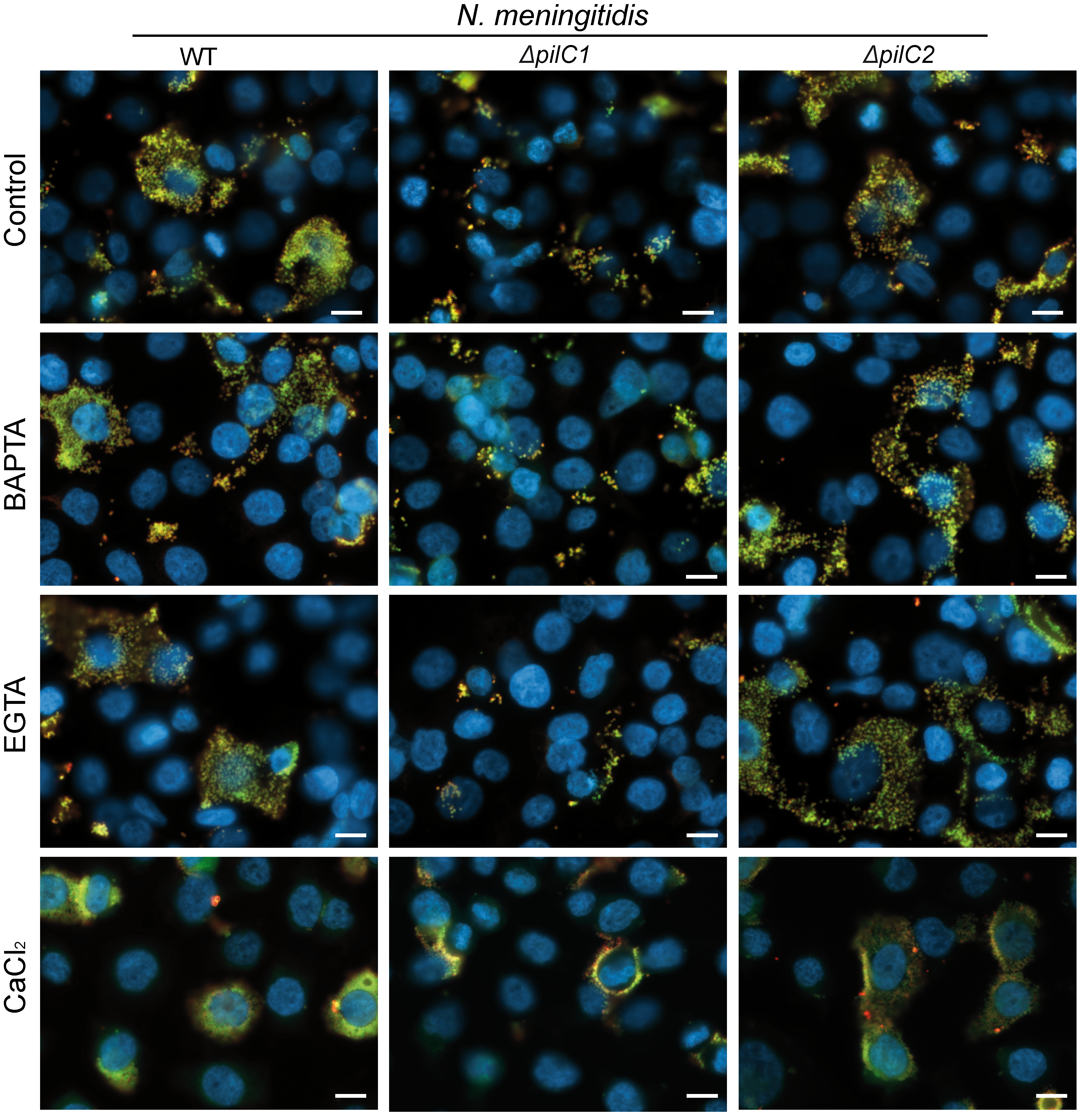

Supplement: Figure S3 — Extracellular Ca2+ concentration has no effect on meningococcal adherence to and subsequent invasion into HBMEC. Host cells pre-treated with extracellular calcium chelators BAPTA and EGTA were infected with three different strains of meningococci (WT, ΔpilC1 and ΔpilC2) with an MOI of 50 for 4 h at 37°C and 5% CO2. Adhered and internalized meningococci to host endothelial cells were visualized by double immunofluorescence staining and fluorescence microscopy as described in Materials and Methods. (TIF) [file pone.0114474.s003.tif]
